# Fatal Ether Operations

**Published:** 1847-06

**Authors:** 


*Fatal Ether Operations.*—The case of lithotomy performed at the Suffolk and Colchester Hospital, proved fatal within twenty-four hours after the operation. We understand, also, that an old gentlemen operated on for some disease of the foot during the last week, by Mr. Travers, died within twenty-four hours of the operation. He never recovered from the stupor caused by the ether.—*Lond. Med. Times.*

---

At the sitting of the French Institute, M. Boussingault gave the details of a number of experiments he made, to prove that salt used with food does not act by uniting with the alimentary matters, and communicating to them properties that render them fitter for digestion but is simply of use in exciting appetite, and thus provoking the greater consumption of food in a given time.—*Dublin Med. Press.*

